# A Microporous Breathable Packaging System for the Postharvest Preservation of Figs (*Ficus carica* L.) in E-Commerce Logistics

**DOI:** 10.3390/foods15132403

**Published:** 2026-07-07

**Authors:** Tong Li, Hongliang Luo, Chenghu Dong, Yang Gao, Cunkun Chen, Na Zhang, Ruixiang Yan

**Affiliations:** 1 Institute of Agricultural Products Preservation and Processing Technology (National Engineering and Technology Research Center for Preservation of Agricultural Products), Tianjin Academy of Agricultural Sciences, Key Laboratory of Storage and Preservation of Agricultural Products, Ministry of Agriculture and Rural Affairs, Tianjin Key Laboratory of Postharvest Physiology and Storage and Preservation of Agricultural Products, State Key Laboratory of Vegetable Biobreeding, Tianjin 300384, China; leetong0606@163.com (T.L.); hongliangpg@163.com (H.L.); dongchenghu2022@126.com (C.D.); chencunkun@126.com (C.C.); 2 College of Light Industry Science and Engineering, Tianjin University of Science & Technology, Tianjin 300222, China; 3 The Research Institute of Forestry and Pomology, Tianjin Academy of Agricultural Sciences, Tianjin 300384, China; 15802265153@163.com

**Keywords:** *Ficus carica* L., preservation, microporous modified atmosphere packaging, shelf life, vibration

## Abstract

Figs are prone to postharvest senescence and decay, causing substantial economic losses. Conventional modified atmosphere packaging is ineffective at elevating the CO_2_ concentration within the package, as the gas exchange rate through the packaging film is insufficient to compensate for the high respiration rate of figs. To explore the effectiveness of packaging in indirectly reducing ethylene accumulation during E-commerce logistics, this study optimized parameters of laser microporous-modified atmosphere packaging (LMMAP) and investigated the effects of LMMAP on the shelf life of figs. Results showed that LMMAP (20 micropores, 300–314 μm in diameter; 30 micropores, 260–270 μm in diameter) effectively regulated the rate of gas exchange across the packaging film under high temperature and vibration stress during transportation. Four days after storage, LMMAP significantly reduced the respiration rate and decay incidence, delayed ethylene release, and maintained the firmness, color and sensory qualities in figs under the same stress conditions. Moreover, LMMAP significantly inhibited the decrease in total soluble solids and titratable acidity content in figs. It also maintains a high antioxidant capacity and reduces the damage to the cell membrane caused by reactive oxygen species. In summary, LMMAP maintains the quality and extends the shelf life of figs during E-commerce logistics.

## 1. Introduction

Figs (*Ficus carica* L.) are one of the oldest domesticated fruit crops, originating from the Middle East and Western Asia, and are now widely cultivated in Mediterranean, subtropical, and tropical regions worldwide [1]. In recent years, global fig production has seen a steady increase, driven by rising consumer demand for fresh and nutritious fruits. As a typical representative of the genus *Ficus*, figs are not only consumed fresh but also processed into dried products, jams, and other value-added goods, making them an economically important crop with sweet and juicy taste, as well as significant nutritional value [2]. Figs usually mature during the high-temperature summer season. As a respiratory climacteric fruit, figs experience a rapid increase in respiration rate and ethylene production after harvesting, leading to ripening and aging within 1 to 2 days, manifested as skin browning, wrinkling, softening, and decay. Moreover, figs have extremely thin skin, soft and juicy flesh, and an open ostiole at the base, making them highly susceptible to mechanical damage, oxidative browning, and pathogen invasion [3]. In addition, with a water content of up to 80–85%, figs undergo strong transpiration after harvest, resulting in peel wilting and shriveling, which significantly reduces their commercial value [4]. These inherent biological and physical attributes render figs far more perishable than typical stone fruits, making postharvest storage and transportation particularly challenging and leading to substantial resource waste and economic losses for producers and retailers.

At present, some methods are used to control the qualities of postharvest figs, including cold storage [5], gaseous ozone [6], irradiation [7] and coating film [8]. However, there are some limitations to these methods. Although low-temperature storage is an effective method to mitigate postharvest respiratory metabolism and extend the shelf-life of fruits and vegetables, the postharvest physiological characteristics vary among different fruit varieties. Inappropriate storage temperatures may induce chilling injury in certain fruits [9]. Furthermore, temperature fluctuations during cold chain transportation can adversely affect fruit quality. In addition, the treatment of irradiation ozone requires energy-intensive and sophisticated equipment. Some edible coatings are operationally complex, and it is essential to ensure consistency across different batches. Modified atmosphere packaging (MAP) is a widely used preservation technique for fruits and vegetables, which delays fruit ripening and senescence by regulating and maintaining the concentrations of O_2_ and CO_2_ in the storage atmosphere [10]. Conventional MAP relies on the inherent gas permeability of the packaging film. For fruits with high respiration rates, this approach may lead to anaerobic conditions caused by excessive CO_2_ accumulation [11]. In contrast, microporous MAP (MMAP) enables more rapid gas exchange with the external environment through microporous film, thereby providing superior preservation performance. The gas composition within MMAP depends largely on packaging area, fruits respiration, and micropore parameters (density, number, and size) [12]. Compared with mechanical cold-needle and hot-needle perforation methods, laser perforation offers higher precision and improved micro-perforation uniformity [13]. Several studies have shown that applying laser microporous modified atmosphere packaging (LMMAP) is effective for postharvest fruit preservation. Lu et al. reported that, compared with non-porous film, the presence of micropores regulated O_2_ and CO_2_ permeability, thereby maintaining the aroma quality of fresh grapes [14]. Zhong et al. found that LMMAP could extend the shelf life of passion fruit to 12 days at 25 °C by regulating water loss and physiological metabolism [15]. Furthermore, for films with micropores arranged at regular intervals, the gas exchange rate is primarily a function of micropore number and size. Based on the postharvest respiration characteristics (the patterns of CO_2_ and O_2_ concentration changes) of different types of fruit, combined with the packaging film parameters, the number and pore size of the micro-perforations in LMMAP can be derived [16]. Studies have shown that LMMAP prepared with 30 μm thick polyacrylamide (5 micropores, 200 μm in diameters) effectively extends the shelf life of cherry tomatoes at room temperature to 11 days [17]. LMMAP prepared with polypropylene (PP) (1 micropore, 50 μm in diameters) and pre-flushed with gas (10% O_2_ + 10% CO_2_ + 80% N_2_) effectively extends the shelf life of fresh-cut peaches under refrigeration to 12 days [18]. LMMAP prepared with PP (60 μm) and pre-flushed with gas (20% CO_2_ + 80% N_2_) effectively extends the shelf life of fresh grapes under refrigeration to 21 days [19]. More importantly, fruits may be exposed to high temperatures and subjected to vibration stress during E-commerce logistics before reaching consumers [20].

The rapid growth of E-commerce has revolutionized the fresh fruit supply chain, with an increasing proportion of figs now being sold directly to consumers through online platforms. Unlike traditional wholesale-retail channels, E-commerce logistics typically involve longer and more fragmented distribution times, exposure to non-refrigerated or fluctuating ambient temperatures, and inevitable mechanical vibration stresses from sorting and transporting packages [21]. These harsh and variable conditions can significantly accelerate the postharvest deterioration of highly perishable figs, leading to a sharp increase in consumer complaints and returns. For instance, vibration stress has been shown to stimulate respiratory metabolism and ethylene production in climacteric fruits, while temperature abuses can promote microbial growth and tissue softening. Conventional MAP are often inadequate to cope with these dynamic stresses. While LMMAP has proven effective under stable storage conditions for several fruits, its specific application and parameter optimization for figs, especially in the context of the challenging E-commerce logistics environment, remain unexplored. Rodov et al. simulated temperature fluctuations during the transportation and storage of bananas and found that LMMAP extended banana shelf life, delayed senescent spotting, and reduced decay rates [22], suggesting a potential strategy. However, the relationship between micropore parameters and the postharvest respiration rate of figs under combined temperature and vibration stress must be investigated in detail.

This study aimed to design the optimal parameters for LMMAP based on the postharvest respiratory characteristics of figs, and systematically analyze the quality changes in figs during the transportation-storage process. The results could provide theoretical support and practical methods for the development of special-purpose LMMAP for fig preservation during E-commerce transportation.

## 2. Materials and Methods

### 2.1. Fruit Materials

A total of 400 fresh fig fruits (*Ficus carica* L. ‘Bojihong’ cultivar) at the seventh maturity grade were harvested on 1 September 2025 from the orchard in Tangshan, Hebei Province, China. Figs with uniformly green skin color, free from pest infestation, disease symptoms, and mechanical damage, were selected for this study. The selected fruits had a firmness of approximately 11.7 N and a total soluble solids (TSSs) content of approximately 10.23%.

### 2.2. Packaging Preparation and Treatment

According to the preliminary experimental results of modified atmosphere and micropore film parameters (Table S1), all the figs were covered with expanded polyethylene (EPE) fruit nets and placed into plastic fresh-keeping boxes (Polypropylene, 20 cm × 15 cm × 6 cm, 1800 mL, CO_2_ TR: 1.13 × 10^−13^ cm^3^·cm/(cm^2^·s·Pa), O_2_ TR: 4.08 × 10^−13^ cm^3^·cm/(cm^2^·s·Pa)). Then, the modified atmosphere packages were gas-flushed with 8% O_2_ + 10% CO_2_ + 82% N_2_. PET/PP composite microporous packaging film (LMMAP, a thickness of 75 μm, CO_2_ TR: 1.13 × 10^−13^ cm^3^·cm/(cm^2^·s·Pa), O_2_ TR: 4.08 × 10^−13^ cm^3^·cm/(cm^2^·s·Pa)) (National Engineering and Technology Research Center for Preservation of Agricultural Products, Tianjin, China) was prepared using a packaging machine (SM-200, Windfor Intelligent Technology Co., Ltd., Tianjin, China) and a laser punching machine (LPG-1100S-D80, Dongguan LLEESUN Power Technology Co., Ltd., Guangzhou, China). Finally, the above-mentioned packages were placed into a corrugated box (BE-shaped, 42 cm × 32 cm × 10 cm, 8000 mL) for further experiments. A schematic diagram of the LMMAP is shown in Figure S1.

The figs were randomly divided into six groups, including (I) CK: EPE nets + corrugated box; (II) RM20: EPE nets + LMMAP (20 micropores, 300–314 μm in diameter) + corrugated box; (III) RM30: EPE nets + LMMAP (30 micropores, 260–270 μm in diameter) + corrugated box; (IV) CK-V: EPE nets + corrugated box + vibration; (V) RM20-V: EPE nets + LMMAP (20 micropores, 300–314 μm in diameter) + corrugated box, + vibration; (VI) RM30-V: EPE nets + LMMAP (30 micropores, 260–270 μm in diameter) + corrugated box + vibration.

### 2.3. Vehicle Vibration Simulation Study

The vehicle vibration simulation study was conducted in accordance with the ISTA 6A standard. The designation of package faces, edges, and corners is illustrated in Figure S2a. The packages of CK-V, RM20-V, and RM30-V groups were placed individually on the vibration table and securely fixed for random vibration testing under simulated road trailer transport conditions. The vibration power spectral density (PSD) profiles are provided in Table 1. The vibration durations were 60 min for face 1, 30 min for face 2, and 30 min for face 5, as specified in Table 2. Subsequently, drop tests were performed on selected faces, edges, and corners, with a total of eight drops (Figure S2b). Following the vibration and drop tests, all groups were stored at 25 ± 2 °C with 60–70% relative humidity for subsequent shelf-life evaluation.

### 2.4. Characterization of Different LMMAP

Optical microscope (OM, SU3500, Hitachi High-Technologies Corporation, Tokyo, Japan) was applied to analyze the morphology and diameters of micropores of LMMAP.

### 2.5. Physiology Indicator Test

O_2_ and CO_2_ concentrations in LMMAP treatments were measured everyday using a combined CO_2_/O_2_ analyzer (Map Scan, Shanghai Jinchuan Electromechanical Technology Co., Ltd., Shanghai, China). The ethylene production and respiration rate of fruits were measured on the basis of Jia et al.’s method [22]. About 300 ± 25 g of figs from each group was placed in LMMAP kept for 2 h, then the ethylene production and respiration rate were determined by gas chromatograph (GC-14, Shimazhu, Tokyo, Japan) and a digital respiration analyzer meter (Check Point 3, PBI-Dansensor, DK-4100, Ringsted, Denmark), respectively. For each treatment, 3 replicate packages were randomly selected at each sampling time point, and 6 fruits from each package were sampled for gas analysis.

### 2.6. Quality Indicator Test

The firmness, decay rate, TSS and titratable acidity (TA) contents of fruits were measured and calculated on the basis of Shi et al.’s method [23]. About 40 figs from each treatment were randomly used to calculate decay rate, and the firmness was measured at three points in the equatorial region of fruits by a texture analyzer (TMS-PRO, Food Technology Corporation, Columbia, SC, USA) with a P/2 steel probe. The TSS content was determined by a protable refractometer (PAL-1, Atago, Tokyo, Japan), and the TA content was measured according to the acid-base neutralization titration method of Ali et al. [24].

### 2.7. Sensory Evaluation Analysis

Surface color was determined by using full automatic Chroma meter (WR10QC, Qingdao Tuoke Instrument Co., Ltd., Qingdao, China). Three points of fruits equator were selected to measure color and calculated L*, a*, and b* values. Flavor characteristics of figs was determined by using an electronic tongue system (TS-5000Z, INSENT, Tokyo, Japan). The sensory quality of figs was evaluated on the basis of De Jesus Filho et al.’s method [25]. The sensory evaluation was conducted by a panel of 12 trained assessors (6 male and 6 female, aged 22–45 years), all of whom were staff and postgraduate students from the Institute of Nutrition and Health, China Agricultural University. All panelists had prior experience in fruit sensory evaluation and were familiar with fig fruit characteristics. Prior to formal evaluation, all panelists participated in two training sessions during which they were familiarized with the sensory attributes of figs and the scoring criteria. All sensory evaluations were conducted in a dedicated sensory testing room maintained at 20 ± 2 °C with white lighting. A 10-point hedonic scale was used to evaluate each attribute. The total sensory score was calculated as the sum of individual attribute scores, with a maximum total score of 40 points. The criteria for sensory evaluation of figs was shown in Table S2. For each treatment, 3 replicate packages were randomly selected at each sampling time point, and 3 fruits from each package were sampled for sensory evaluation analysis.

### 2.8. Antioxidant and Oxidative Browning-Related Enzymes Activities Test

The contents of superoxide dismutase (SOD), malondialdehyde (MDA), H_2_O_2_, Polyphenol oxidase (PPO), peroxidase (POD) and O_2_^−^· production rate were measured using different commercial kits following the instructions (Nanjing Jiancheng Bioenginerring Institute, Nanjing, China). For each treatment, 3 replicate packages were randomly selected at each sampling time point, and 3 fruits from each package were sampled for biochemical measurements.

### 2.9. Statistical Analysis

All experiments were carried out at least in triplicate and results were expressed as the mean ± standard deviation. Statistical analysis was performed using ANOVA-Tukey analysis, or Student’s *t*-test (GraphPad Prism, version 8.0.6, San Diego, CA, USA). Sensory score and taste characteristic analysis was performed and analyzed using Origin (Version 9, Northampton, MA, USA). Correlation analysis and mantel test was using chiplot online tool (http://www.chiplot.online/#, URL (accessed on 1 May 2026)). Differences between means were considered to be significant when the *p* was <0.05.

## 3. Results

### 3.1. Preparation and Characterization of LMMAP

**Figure 1:**
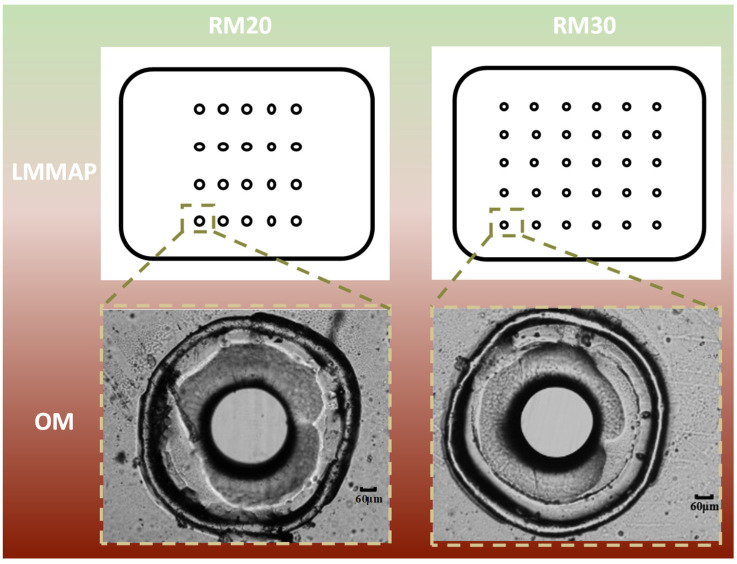
Characterization of different LMMAP treatments. Scale bar = 60 μm.

The optimal gas composition in LMMAP was screened through preliminary experiments (Figures S3 and S4). Firstly, the CO_2_ concentration was set at 0.03%, and a range of O_2_ concentrations (5, 10, 20, 30, 40%) were tested. By measuring the physiological and biochemical indices of post-harvest figs, the optimal O_2_ concentration was determined to be 10%. Secondly, with the O_2_ concentration fixed at 10%, different CO_2_ concentrations (3, 6, 9, 12, 15, 20%) were evaluated. The results showed that the most suitable gas ratio was 10%O_2_ + 6%CO_2_ + 84%N_2_ in LMMAP. In addition, based on Knudsen diffusion theory, the fig respiration model and the LMMAP gas model are integrated to determine the gas exchange rate of the packaging system at a specified time, enabling the prediction of time-dependent changes in gas concentrations within the package (Equations (S1) and (S2)). The gas diffusion rate through the micropores is described by Fick’s law of diffusion, which is proportional to the total micropore area and the gas concentration gradient. The gas permeation rate through the film is described by Henry’s law, which depends on the film material’s permeability coefficient and the effective film area. As shown in Figure 1, the OM analysis indicated that the diameter of micropores was ≈300 μm of RM20, and the diameter of micropores was ≈260 μm of RM30. This indicated that prepared LMMAP successfully achieved the optimal microporous parameters as derived from the respiration rate of figs stored under ambient temperature conditions (Table S1).

### 3.2. LMMAP Reduces Post-Harvest Physiological Activity of Figs

**Figure 2:**
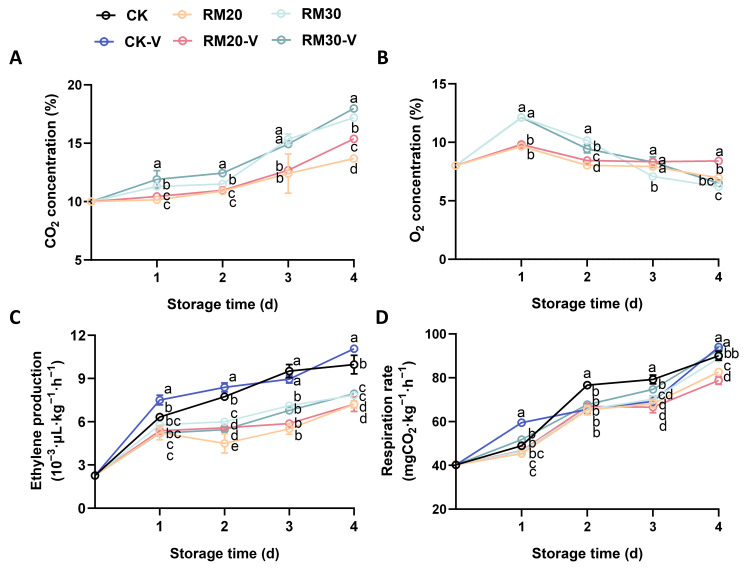
Effects of LMMAP treatment on internal O_2_ concentration (**A**), CO_2_ concentration (**B**), ethylene production (**C**), and respiration rate (**D**) of figs during storage. Different letters indicate significant differences among all the groups (*p* < 0.05).

In contrast to conventional MAP, MMAP achieves an optimal gas environment for fruits and vegetables by controlling the exchange of O_2_ and CO_2_ between the package interior and the external atmosphere [26]. The actual transit and retail display time for figs via E-commerce platforms is typically 3 to 4 days from harvest to consumer. Accordingly, we set the storage period at 4 days to adequately represent commercial logistics and household storage conditions. Figure 2A,B shows the evolution of the gas concentrations during storage. In all groups, the O_2_ content within the packages exhibited a significant increase on the first day of storage, after which it gradually declined. Conversely, the CO_2_ concentration showed a sustained upward trend, with a marked increase observed on the final day of storage. This phenomenon can be attributed to the rapid ripening and senescence of postharvest figs, as respiratory metabolism consumes O_2_ and produces CO_2_. Compared with the vibration-stressed groups, both RM20 and RM30 effectively reduced CO_2_ accumulation within the packages and maintained relatively stable O_2_ concentrations. This indicates that LMMAP is effective in controlling CO_2_ accumulation and reducing the risk of anaerobic stress in figs.

The fig is a typical climacteric fruit. After harvest, it undergoes a sudden increase in respiration rate and releases a large amount of ethylene gas, which triggers self-ripening, followed by rapid senescence [27]. As shown in Figure 2C,D, the ethylene production and respiration rate of figs exhibited a gradual increase during storage, and reached a peak on day 4. All of the LMMAP groups showed significantly lower respiration intensity and ethylene production compared to the CK group. At the end of storage, the respiration intensity in the RM20 and RM30 treatment groups was reduced by 44.10% and 39.86%, respectively, relative to the CK group, while the ethylene production rate was reduced by 27.69% and 21.45%, respectively. Furthermore, we found that vibration stress accelerates the ripening and senescence of figs. Compared with the CK group, the CK-V group exhibited higher ethylene production and postharvest respiration rates. However, treatment with LMMAP effectively mitigated fruit senescence induced by vibration and high-temperature stress by maintaining a balanced gas exchange between the inside and outside of the package. Above all, these results demonstrate that LMMAP exhibited a positive effect on the preservation of postharvest figs during transportation under high temperature and vibration stress.

### 3.3. LMMAP Enhances Post-Harvest Storage Quality of Figs

**Figure 3:**
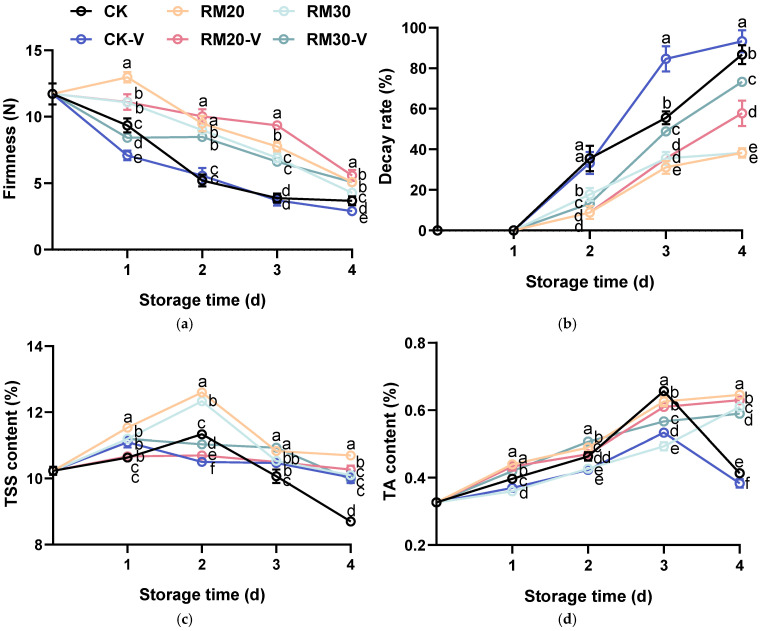
Effects of LMMAP treatment on firmness (**a**), decay rate (**b**), TSS content (**c**), and TA content (**d**) of figs during storage. Different letters indicate significant differences among all the groups (*p* < 0.05).

Firmness and decay rate serve as key indicators of fruit freshness. As shown in Figure 3a,b, the firmness of figs exhibited a decreasing trend, whereas the decay rate showed an increasing trend during the storage. Compared with the CK group, LAMMAP treatment significantly decreased the decay rate of figs, with RM20 exhibiting a better effect than RM30. As expected, vibration stress resulted in an increase in fruit decay rate. Valero et al. [28] reported that fruit softening was correlated with bruise susceptibility. In this study, LMMAP treatment preserved the fresh firmness of figs, indicating that LMMAP treatment could delay textural deterioration of fig fruit during logistics.

Furthermore, TSS and TA contents influence fruit acidity and flavor, serving as important indicators of fruit quality. TSS content exhibited a trend of rising followed by declining (Figure 3c). At the end of storage, RM20 treatment maintained a higher TSS content (10.70%). As shown in Figure 3d, for both the CK and CK-V groups, the values reached their peak on day 3 of storage and subsequently decreased. While the LMMAP treatment group showed an increasing trend in TA content throughout the storage period. Under both non-vibration and vibration stress conditions, RM20 demonstrated superior preservation of TA content compared with RM30.

### 3.4. LMMAP Enhances Post-Harvest Sensory Quality of Figs

**Figure 4:**
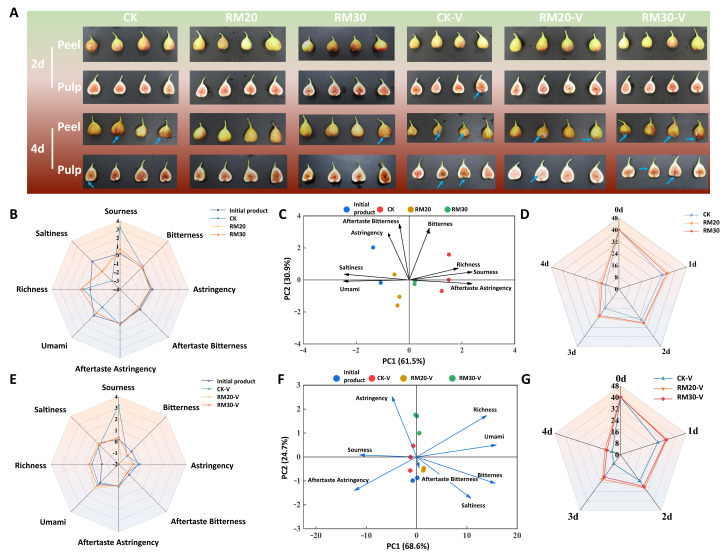
Effects of LMMAP treatment on appearance (**A**), electronic tongue response value (**B**,**E**), electronic tongue PCA analysis (**C**,**F**), and sensory score (**D**,**G**) of figs during storage. The blue arrow indicates the decay and deterioration of the (**A**).

The change in color represents a key visual indicator of postharvest fruit ripening and senescence, influencing consumer acceptance while also reflecting the underlying physiological and biochemical status of the fruit [1]. As shown in Table 3, the a* and b* values of figs increased, while the L* value decreased, indicating a shift toward red and yellow and a gradual loss of brightness in the peel of figs during shelf life. The RM20 group effectively delayed this color transition and preserved peel brightness, whereas vibration stress accelerated the color change. These findings are consistent with Figure 4A. The CK group showed bottom rot, mold, and juice exudation (blue arrow), while LMMAP significantly reduced fruit decay, with RM20 being the most effective. As expected, vibration stress exacerbated fruit deterioration.

Flavor analysis showed that the CK group had higher sourness, astringency, and bitterness, while the RM20 group had higher umami and saltiness and the RM30 group had higher richness, indicating distinct taste differences in LMMAP. At the end of storage, the CK group still exhibited higher sourness, astringency, and bitterness than the LMMAP group (Figure 4B). As shown in Figure 4C, First Principal Component (PC1) and Second Principal Component (PC2) accounted for 92.4% of the total variance, effectively capturing the taste characteristics and changes during storage. On day 4, the CK group positively correlated with sourness, astringency, and bitterness, whereas RM20 positively correlated with umami and saltiness, and RM30 positively correlated with richness. These PCA results aligned well with the electronic tongue data. Furthermore, sensory analysis of postharvest figs revealed that after three days of storage, the sensory score of the CK group fell below 20, whereas that of the LMMAP group was significantly higher, indicating that these figs still retained commercial value (Figure 4D). As shown in Figure 4E–G, RM20-V showed similar flavor characteristics to the RM20 group. PC1 and PC2 accounted for 68.6% and 24.7% of the variance, respectively, effectively representing the overall flavor profile of the fig fruit. On storage day 4, CK-V positively correlated with sourness and astringency, whereas RM20-V positively correlated with saltiness, umami, and bitterness, as well as RM30-V positively correlated with bitterness and richness. This indicates that vibration stress during E-commerce logistics may affect fig flavor by accelerating fruit ripening and senescence.

### 3.5. LMMAP Regulates Antioxidant and Oxidative Browning-Related Enzymes Activities of Post-Harvest Figs

**Figure 5:**
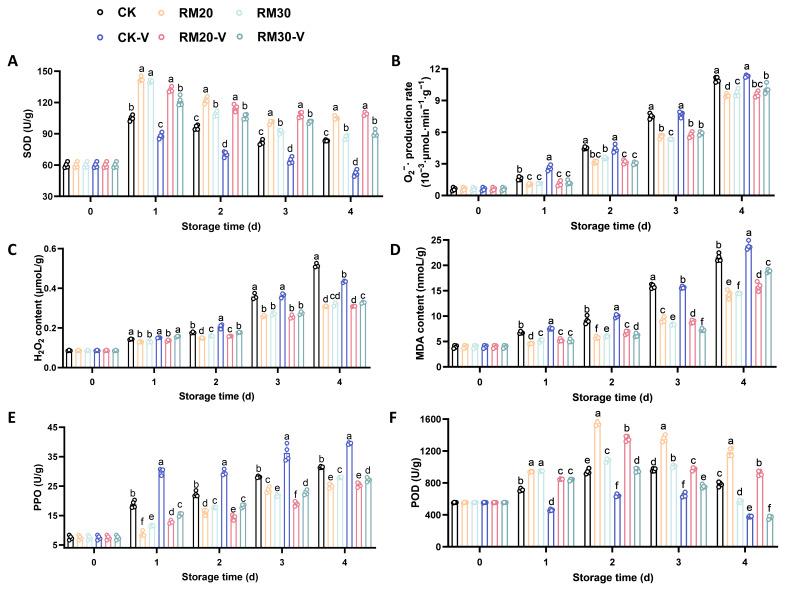
Effects of LMMAP treatment on SOD (**A**), O_2_^−^· production rate (**B**), H_2_O_2_ content (**C**), MDA content (**D**), PPO (**E**) and POD (**F**) of figs during storage. (a–f). indicate significant differences among treatment groups at the same storage time (*p* < 0.05).

As outlined in Figure 5, after CO_2_ peaked in the LMMAP system (day 4), the contents of O_2_^−^·, MDA and H_2_O_2_ increased sharply, which may have been caused by the accumulation of large amounts of alcohol resulting from harmful anaerobic respiration, leading to cell membrane damage. Furthermore, SOD is a key enzyme for ROS scavenging, PPO is the primary catalyst of enzymatic browning, and POD contributes to maintaining ROS homeostasis by scavenging H_2_O_2_ and lipid hydroperoxides [29]. The senescence of figs is closely related to the activity levels of these enzymes. The SOD and POD activities in the CK and CK-V groups exhibited a trend of initially increasing and then decreasing, peaking on day 4 of storage. Under both vibration and non-vibration conditions, the SOD and POD activities in the RM20 group were significantly higher than those in the CK group. Furthermore, PPO catalyzes the oxidation of plant endogenous phenolics to quinones in the presence of oxygen, leading to browning [30]. Vibration stress and high temperature increased PPO activity in figs, while LMMAP treatment significantly alleviated this increase. Above all, LMMAP reduced excessive ROS accumulation by regulating postharvest respiration and the rate of gas exchange across the film, thereby preserving high antioxidant enzyme activities in the fruit.

### 3.6. Analysis of LMMAP Treatment on the Correlation Between Quality Attributes and Physiological Indices of Figs

**Figure 6:**
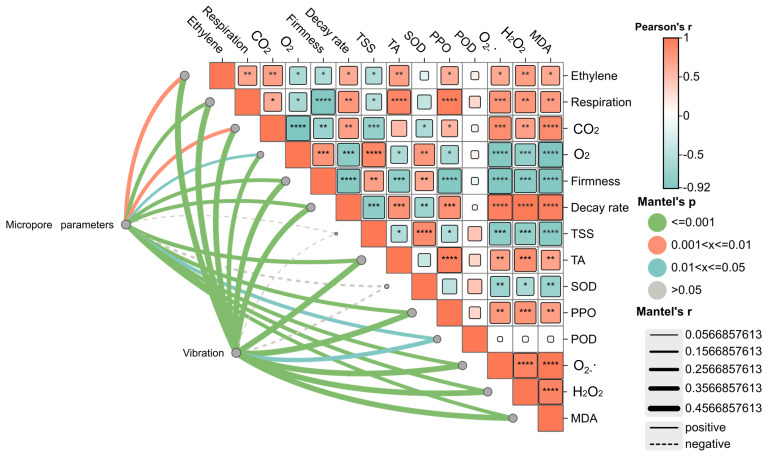
Correlations between changes in ethylene production, respiration rate, firmness, decay rate, the concentration of O_2_ and CO_2_, nutrient content, and enzyme activities in figs. Mantel tests were performed to evaluate the relationships among LMMAP, vibration, and micropore parameters during storage process. The heatmap was performed with Pearson’s correlation coefficients of different indices in the RM20 group. Red (+1) and blue (−1) were positively and negatively correlated with different indicators, respectively. The edge color and width represent the statistical significance of Mantel’s *p* and the magnitude of Mantel’s r, respectively. *, **, and *** indicate correlations at *p* < 0.05, *p* < 0.01, and *p* < 0.001, respectively. **** notation indicates a statistical significance level of *p* < 0.0001.

To further explore the effects of LMMAP on the physiological quality of figs during storage, correlations between ethylene production, respiration rate, firmness, decay rate, the concentration of O_2_ and CO_2_, nutrient content, and enzyme activities were analyzed. The Pearson correlation coefficient (R) and mantest analysis were used to construct a correlation heatmap (Figure 6). In RM20 group, the results indicated that the ethylene release and respiration rate were positively correlated with CO_2_ concentration, O_2_^−^· production rate, the contents of H_2_O_2_, MDA and TA, the activities of PPO and POD, and decay rate. And the ethylene release and respiration rate were negatively correlated with firmness and O_2_ concentration. Furthermore, mantel tests revealed that micropores parameters (RM20) and vibration stress (RM20-V) were strongly associated with ethylene production, respiration rate, firmness, decay rate, the concentration of O_2_ and CO_2_, nutrient content, and enzyme activities. However, no significant associations were found with TSS or SOD content.

## 4. Discussion

Fig fruits are thin-skinned, juicy, sweet, seedless, and possess antioxidant properties. However, their thin skin and high moisture content lead to intense postharvest respiratory metabolism and high susceptibility to pathogen infection, resulting in rapid decay, which results in significant financial losses [31]. Conventional MAP relies solely on the intrinsic permeability of the film, which is often insufficient for high-respiring fruits like figs, leading to anaerobic respiration and CO_2_ injury [3]. The gas transmission rate of LMMAP is governed by the combined contribution of intact film permeation and Knudsen diffusion theory through micropores. In this study, the number of micropores was the primary variable modulating total gas permeability, as the film area and material were kept constant. The CK group exhibited a sharp CO_2_ peak accompanied by elevated ethanol production (indicated by the concurrent rise in MDA and H_2_O_2_), a classic symptom of fermentative metabolism. In contrast, LMMAP with 20 micropores allowed adequate O_2_ ingress and CO_2_ egress, maintaining gas concentrations within the tolerance range of fig physiology. As shown in Figure 2 and Figure 3, an increase in ethylene production would result in a faster rate of decay and fruit softening. This finding is supported by Huan et al., who reported that ethylene accumulation was highest and firmness was lowest during the late stages of storage at room temperature [32]. Furthermore, it has been suggested that plant cell wall metabolism regulates ethylene at the transcriptional level [33]. We also found that vibration stress enhanced ethylene production and respiration rate in figs, probably due to the activation of key ethylene biosynthetic enzymes triggered by mechanical stress, which accelerated fruit ripening and senescence [34]. Above all, with 20 micropores (260 μm diameter), the system achieved an equilibrium atmosphere that sufficiently suppressed respiratory metabolism without inducing anaerobic stress, which is consistent with the review on modified atmosphere packaging for tropical fruits by Paul and Pandey [35].

As shown in Figure 3c,d, the TSS content exhibited an initial increase followed by a subsequent decrease. This may be attributed to the postharvest ripening of figs, during which starch is broken down into soluble sugars such as glucose, fructose, and sucrose by amylase, leading to an increase in TSS. In the later stages of storage, soluble sugars and organic acids serve as substrates and are metabolized into CO_2_ and H_2_O via glycolysis and the tricarboxylic acid cycle, accompanied by energy release. When the rate of respiratory consumption exceeds the rate of sugar production or conversion, TSS content begins to decline [36]. TA content exhibited a declining trend throughout the entire storage period. This may be attributed to the degradation of macro-molecular carbohydrates stored in figs into soluble sugars and organic substances, driven by the increase in respiration [22]. In addition, the darkening and reddening of fig peel are key characteristics of postharvest senescence and oxidation, accompanied by gradual chlorophyll degradation and increasing trends in a* and b* values. Studies have shown that MMAP may delay the reddening of the peel by affecting pigment metabolism in peel cells, such as the synthesis of anthocyanins mediated by the phenylalanine pathway [37]. As presented in Table 3 and Figure 4, under high-temperature and vibration stress conditions, both RM20 and RM20 maintained better peel color in the fruit. POD is another important enzyme that participates in chlorophyll degradation by interacting with chlorophyll genes. Studies have shown that MAP can upregulate the expression of POD genes, thereby inducing an increase in POD activity. This consequently delays browning and oxidation, and enhances the antioxidant defense system [38]. Compared with the CK and CK-V groups, the LMMAP group exhibited a reduced decline in POD activity (Figure 5F). Furthermore, the gas environment within LMMAP is unfavorable for the growth and reproduction of certain spoilage microorganisms, thereby reducing their growth rate. In addition, the buffering effect and modified atmosphere of LMMAP reduce the availability of nutrients that can be utilized by microorganisms, indirectly limiting their nutritional sources [39]. The activity and survival of fresh plant cells are closely related to the dynamic balance of reactive oxygen species (ROS) [40]. Excessive ROS attack the cell wall, causing membrane lipid peroxidation and leading to fruit softening [41]. The elimination of ROS in plants is mainly regulated by antioxidant enzyme systems and non-enzymatic antioxidants [42]. As shown in Figure 5, the sharp increase in MDA, H_2_O_2_, and O_2_^−^· following the CO_2_ peak can be explained by excessive anaerobic alcohol production, which directly links to cell membrane damage, water loss, and reduced firmness. LMMAP better maintained SOD and PPO activities and enhanced fruit antioxidant activity. Above all, the efficacy of LMMAP in reducing quality loss in fruits lies in its ability to synchronously modulate multiple interconnected physiological pathways, which is consistent with the results of Gun et al. [43] and Liu et al. [44].

In addition, there are few studies that investigate the correlation between environmental factors such as vibration and micropores parameters and quality indicators. In this study, we examined the relationship between the physiological metabolism and quality of postharvest figs in RM20 treatment, as well as the effects of varying micropore parameters (RM20) and vibration stress (RM20-V). No correlation was found between these factors and TSS or SOD contents (Figure 6). This is because micropore parameters mainly regulate gas concentrations, while vibration stress directly causes physical damage and metabolic disruption. TSS levels during long-term storage depend largely on respiratory substrate consumption and water loss, which are only indirectly influenced by these treatments. Meanwhile, SOD activity initially increases to counteract oxidative stress but declines under sustained damage due to impaired enzyme synthesis. Such a complex, time-dependent response is unlikely to correlate simply with a single micropore parameter or vibration intensity.

E-commerce logistics of fresh figs impose unique stresses, including high temperature, mechanical vibration, and unpredictable transit times. Our results demonstrate that LMMAP with 20 micropores effectively mitigates the negative effects of vibration stress by maintaining a stable internal atmosphere that buffers against transient increases in respiration rate triggered by mechanical disturbance. This buffering capacity is particularly valuable for practical supply chains, where temperature and handling conditions are often suboptimal. In addition, laser perforation inevitably alters the mechanical properties of the packaging film. Although the micropores (260 μm) are relatively small compared to the total film area, stress concentration around the pore edges may reduce tensile strength and puncture resistance, particularly under vibration stress during logistics. In our study, no film rupture was observed in any LMMAP treatment, suggesting that the selected pore size and number remained within the mechanical tolerance of the film.

LMMAP demonstrates considerable potential for preserving postharvest figs during E-commerce logistics. Notably, while laser perforation offers advantages in precision, high-speed operation, and consistent quality, it entails higher capital investment than mechanical perforation methods. The integrity of package seals under vibration stress during transportation requires further validation. Additionally, condensation-induced pore blockage, a known challenge in high-moisture produce packaging, may compromise gas exchange. The use of absorbent pads could be considered to mitigate this issue. Furthermore, given that different fig cultivars exhibit variations in respiration rate and ethylene production, micropore parameters may require cultivar-specific optimization.

However, several limitations of this study should be acknowledged. First, the experiments were conducted under simulated constant-temperature conditions, whereas real-world logistics involve temperature fluctuations that may nonlinearly affect film permeability and fruit respiration rates. Second, it should be acknowledged that the present study did not include a conventional MAP as a control group. Future work should include a systematic comparative evaluation of LMMAP versus conventional MAP, which would further substantiate the superiority of the LMMAP approach in the context of E-commerce logistics. Third, for practical operational convenience, we selected only RM20 and RM30 from the full range of predicted microporous configurations for experimental validation. Future studies should systematically investigate the untested configurations (RM10, RM40, RM50) to establish a comprehensive parameter optimization framework for fig LMMAP under dynamic logistics conditions. In addition, future research should address these gaps by: (i) validating LMMAP performance under real e-commerce supply chains with dynamic temperature profiles and (ii) developing adaptive or intelligent packaging systems equipped with gas sensors for real-time monitoring. Such efforts are essential to facilitate industrial translation and reduce postharvest losses in fresh-fruit e-commerce.

## 5. Conclusions

This study demonstrates that LMMAP with 20 micropores (260 μm diameter) effectively extends the shelf life of figs during E-commerce logistics. The package maintains fruit quality by regulating gas exchange under transport stress, which in turn suppresses ethylene production, enhances antioxidant enzyme activities, preserves TSS and TA contents, and reduces ROS accumulation, thereby delaying senescence and decay. Future studies should examine the long-term effects of LMMAP on flavor-related volatile compounds, and develop adaptive packaging systems capable of responding to dynamic temperature conditions along the supply chain.

## Figures and Tables

**Table 1:** Conditions of simulated road trailer test.

Frequency (Hz)	PSD Grade (g^2^/Hz)
1	0.0007
3	0.02
5	0.02
7	0.001
12	0.001
15	0.004
24	0.004
28	0.001
36	0.001
42	0.003
75	0.003
200	0.000004

**Table 2:** Parameters of simulated road trailer test.

Falling Order	Height (mm)	Location	Time (min)
1	460	1	60
2	460	3	30
3	460	5	30

**Table 3:** Effect of LMMAP on the color difference in figs during shelf life.

Indicators	Groups	Storage Time (d)				
		0	1	2	3	4
L*	CK	74.99 ± 0.99 ^a^	72.32 ± 0.51 ^b^	71.77 ± 0.38 ^c^	70.71 ± 0.49 ^b^	68.75 ± 0.47 ^c^
	RM20	74.99 ± 0.99 ^a^	74.15 ± 0.64 ^a^	73.64 ± 0.56 ^a^	72.03 ± 0.40 ^a^	71.19 ± 0.40 ^a^
	RM30	74.99 ± 0.99 ^a^	73.40 ± 0.36 ^a^	72.73 ± 0.27 ^b^	71.82 ± 0.26 ^a^	70.0 ± 0.28 ^b^
	CK-V	74.99 ± 0.99 ^a^	71.93 ± 0.65 ^c^	70.30 ± 0.18 ^b^	68.68 ± 0.31 ^c^	64.97 ± 0.46 ^c^
	RM20-V	74.99 ± 0.99 ^a^	73.81 ± 0.35 ^a^	72.86 ± 0.43 ^a^	71.87 ± 0.23 ^a^	71.37 ± 0.34 ^a^
	RM30-V	74.99 ± 0.99 ^a^	73.08 ± 0.51 ^b^	72.44 ± 0.56 ^a^	71.11 ± 0.34 ^b^	69.50 ± 0.40 ^b^
a*	CK	−5.58 ± 0.55 ^a^	−3.47 ± 0.31 ^b^	−1.97 ± 0.31 ^b^	1.21 ± 0.15 ^b^	2.96 ± 0.31 ^b^
	RM20	−5.58 ± 0.55 ^a^	−3.45 ± 0.35 ^b^	−2.91 ± 0.26 ^c^	−2.32 ± 0.29 ^c^	−2.02 ± 0.33 ^c^
	RM30	−5.58 ± 0.55 ^a^	−3.30 ± 0.16 ^b^	−2.83 ± 0.21 ^c^	−1.91 ± 0.17 ^b^	−1.69 ± 0.13 ^c^
	CK-V	−5.58 ± 0.55 ^a^	−0.95 ± 0.59 ^a^	0.08 ± 0.29 ^a^	2.01 ± 0.28 ^a^	4.13 ± 0.22 ^a^
	RM20-V	−5.58 ± 0.55 ^a^	−4.72 ± 0.26 ^c^	−4.20 ± 0.40 ^d^	−3.80 ± 0.37 ^d^	−1.15 ± 0.26 ^d^
	RM30-V	−5.58 ± 0.55 ^a^	−4.41 ± 0.25 ^c^	−3.44 ± 0.13 ^c^	−2.49 ± 0.35 ^c^	−0.76 ± 0.40 ^c^
b*	CK	39.56 ± 0.49 ^a^	43.43 ± 0.48 ^a^	43.76 ± 0.36 ^a^	45.20 ± 0.31 ^a^	46.11 ± 0.48 ^a^
	RM20	39.56 ± 0.49 ^a^	40.52 ± 0.28 ^b^	41.05 ± 0.26 ^c^	42.40 ± 0.30 ^c^	42.92 ± 0.33 ^b^
	RM30	39.56 ± 0.49 ^a^	40.63 ± 0.15 ^b^	41.44 ± 0.08 ^b^	42.88 ± 0.12 ^b^	43.12 ± 0.12 ^b^
	CK-V	39.56 ± 0.49 ^a^	43.12 ± 0.41 ^a^	45.43 ± 0.32 ^a^	46.17 ± 0.43 ^a^	48.50 ± 0.35 ^a^
	RM20-V	39.56 ± 0.49 ^a^	40.92 ± 0.38 ^c^	41.2 ± 0.37 ^c^	41.85 ± 0.44 ^c^	42.70 ± 0.23 ^c^
	RM30-V	39.56 ± 0.49 ^a^	41.46 ± 0.32 ^b^	42.50 ± 0.38 ^b^	42.85 ± 0.22 ^b^	43.22 ± 0.32 ^b^

a–d indicate significant differences between groups for the same indicator (*p* < 0.05).

## Data Availability

The original contributions presented in this study are included in the article. Further inquiries can be directed to the corresponding authors.

## Supplementary Materials

The following supporting information can be downloaded at https://www.mdpi.com/article/10.3390/foods15132403/s1, Figure S1: Schematic diagram of the LMMAP; Figure S2: Diagram of ISTA 6A test; Figure S3: The impact of varying oxygen concentrations on the taste of figs; Figure S4: Effects of different carbon dioxide concentrations on sensory score of figs; Table S1: Predicted micropore parameters of LMMAP for fig preservation under ambient temperature conditions; Table S2: Criteria for sensory evaluation of figs.

## Abbreviations

The following abbreviations are used in this manuscript:

CK	Control group
CK-V	Control group with vibration
MAP	Modified atmosphere packaging
MMAP	Microporous modified atmosphere packaging
LMMAP	Laser microporous modified atmosphere packaging
PC1	First Principal Component
PC2	Second Principal Component
PP	Polypropylene
EPE	Expanded polyethylene
OM	Optical microscope
TSS	Total soluble solids content
TA	Titratable acidity
MDA	Malondialdehyde
PPO	Polyphenol oxidase
POD	Peroxidase
RM20	20 micropores
RM20-V	20 micropores with vibration
RM30	30 micropores
RM30-V	30 micropores with vibration
