# Supplementary material for: A Microporous Breathable Packaging System for the Postharvest Preservation of Figs (Ficus carica L.) in E-Commerce Logistics
Source: Foods. 2026 Jul 7;15(13):2403. doi: 10.3390/foods15132403 (PMC13361534; doi:10.3390/foods15132403)
Supplement: Supplementary file 1 [file foods-15-02403-s001.zip › foods-4332599-supplementary.pdf]

Supporting Information

A Novel Microporous Breathable Packaging System for the Postharvest Preservation of Figs (*Ficus carica* L.) in E-Commerce Logistics

Tong Li [1], Hongliang Luo [1,2], Chenghu Dong [1], Yang Gao [3], Cunkun Chen [1], Na Zhang [1,*] and Ruixiang Yan [2,*]

[1] Institute of Agricultural Products Preservation and Processing Technology (National Engineering and Technology Research Center for Preservation of Agricultural Products), Tianjin Academy of Agricultural Sciences, Key Laboratory of Storage and Preservation of Agricultural Products, Ministry of Agriculture and Rural Affairs, Tianjin Key Laboratory of Postharvest Physiology and Storage and Preservation of Agricultural Products, State Key Laboratory of Vegetable Biobreeding, Tianjin 300384, China; leetong0606@163.com (T.L.); hongliangPG@163.com (H.L.); dongchenghu@sina.com (C.D.); chencunkun@126.com (C.C.); wuaide-hua@163.com (N.Z.)

[2] College of Light Industry Science and Engineering, Tianjin University of Science & Technology, Tianjin 300222, China; yrxan@163.com (R.Y.)

[3] The Research Institute of Forestry and Pomology, Tianjin Academy of Agricultural Sciences, Tianjin, China; 15802265153@163.com (Y.G.)

* Correspondence: wuaidehua@163.com (N.Z.); yrxan@163.com (R.Y.)

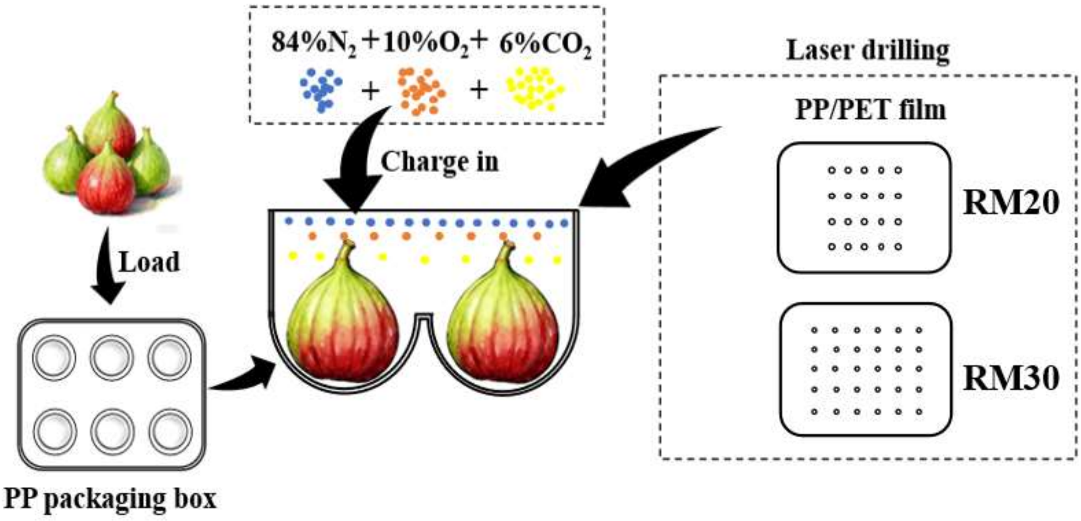

Figure S1 Schematic diagram of the LMMAP.

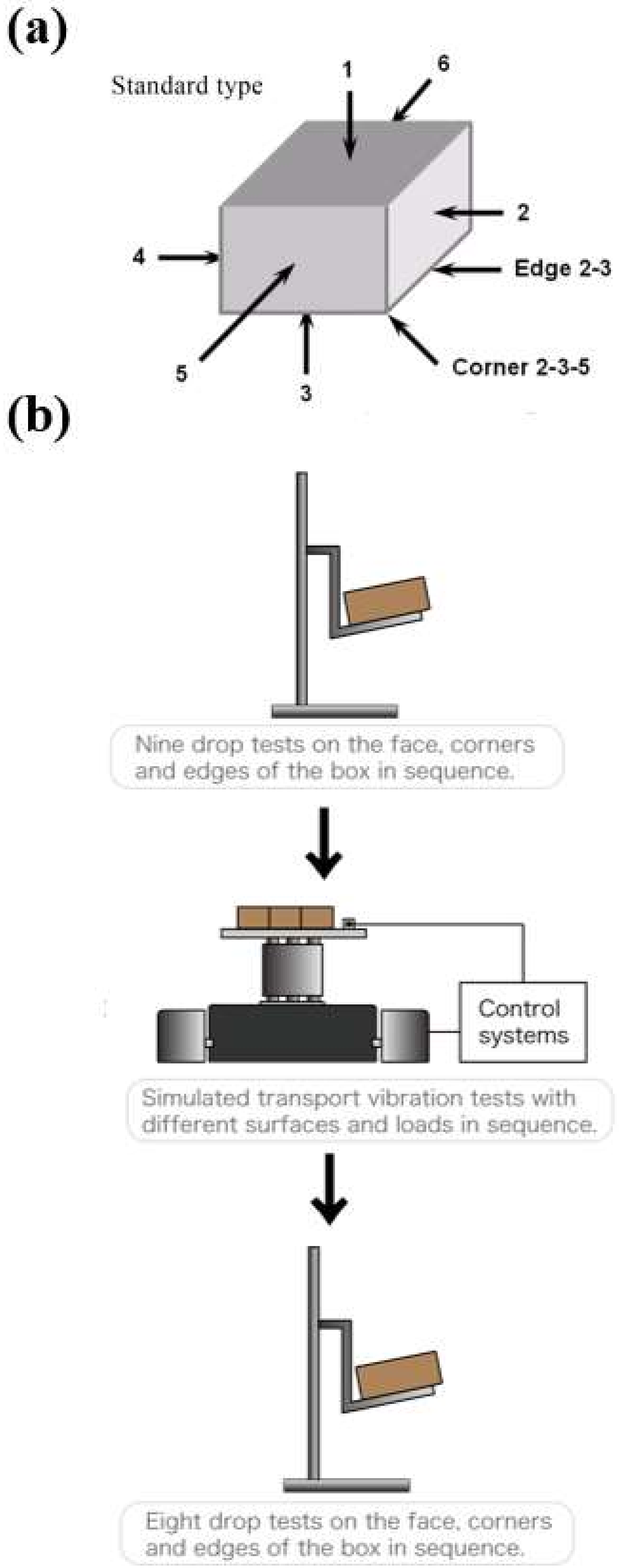

Figure S2 Diagram of ISTA 6A test. (a) Marking of box faces, corners and edges; (b) Test standards for ISTA 6A

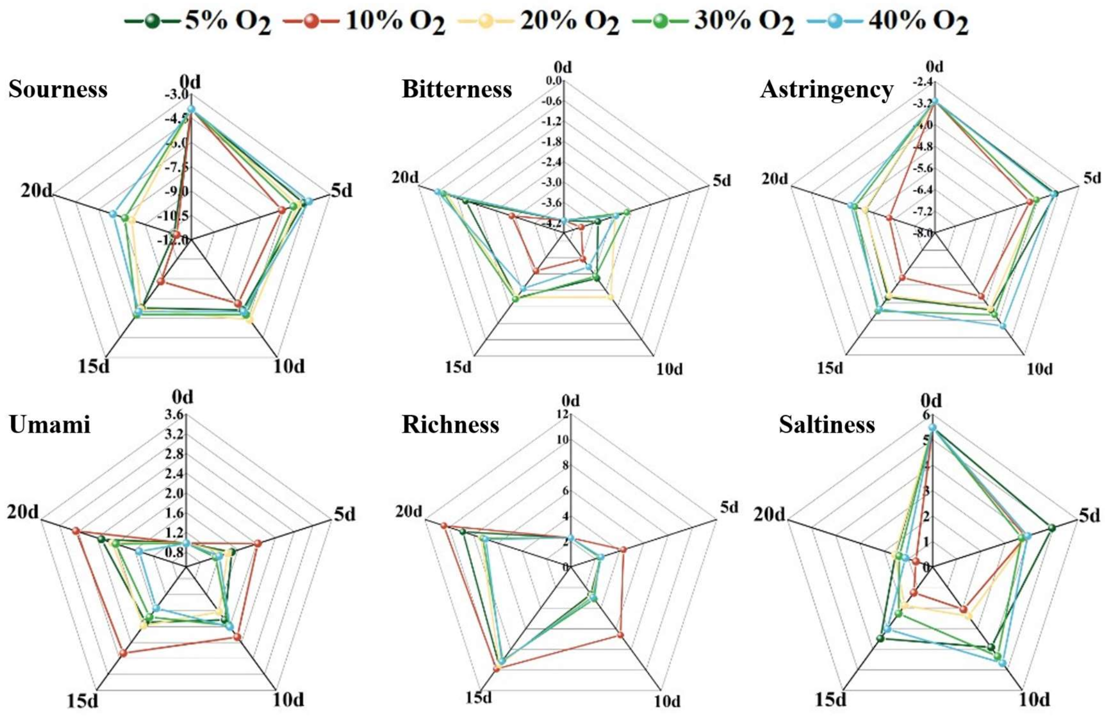

Figure S3 The impact of varying oxygen concentrations on the taste of figs

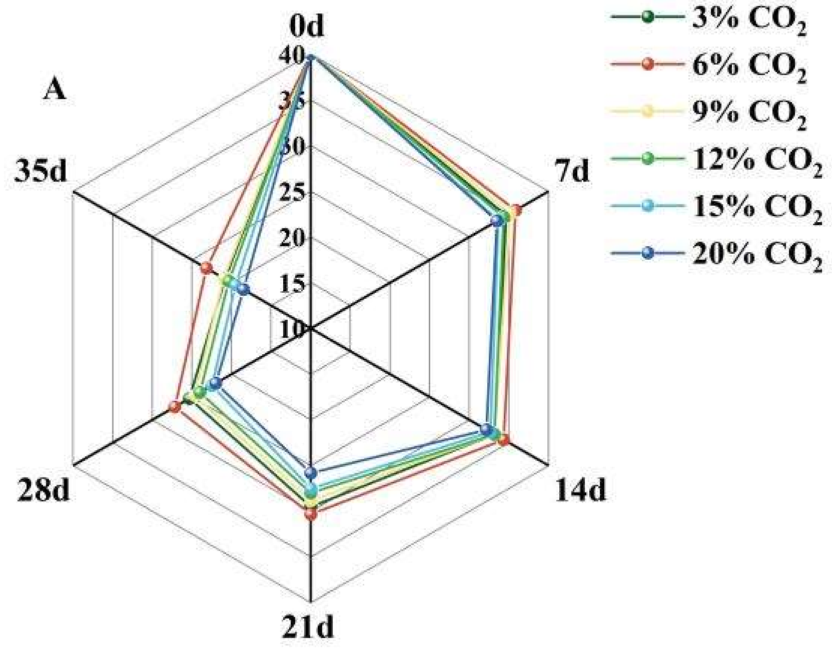

Figure S4 Effects of different carbon dioxide concentrations on sensory score of figs

Fi

Equation (S1):

$$Nd^{3}_{(O_2)}=\frac{12\,\dfrac{V_{mO_2}[O_2]_{zj}}{K_{mO_2}+[O_2]_{zj}\left(1+\dfrac{[CO_2]_{zj}}{K_i o_2}\right)}Lm-12P_{M,O_2}\times(P_{out,O_2}-\dfrac{P_{in,N_2}}{1-[O_2]_{zj}-[CO_2]_{zj}}[O_2]_{zj})\times A}{\pi A(P_{out,O_2}-\dfrac{P_{in,N_2}}{1-[O_2]_{zj}-[CO_2]_{zj}}[O_2]_{zj})\times\sqrt{\dfrac{8}{\pi MO_2RT}}}$$

Equation (S2):

$$Nd^{3}_{(CO_2)}=\frac{-12\,\dfrac{V_{mCO_2}[O_2]_{zj}}{K_{mCO_2}+[O_2]_{zj}\left(1+\dfrac{[CO_2]_{zj}}{K_i CO_2}\right)}Lm-12P_{M,CO_2}\times(P_{out,CO_2}-\dfrac{P_{in,N_2}}{1-[O_2]_{zj}-[CO_2]_{zj}}[CO_2]_{zj})\times A}{\pi A(P_{out,CO_2}-\dfrac{P_{in,N_2}}{1-[O_2]_{zj}-[CO_2]_{zj}}[CO_2]_{zj})\times\sqrt{\dfrac{8}{\pi MCO_2RT}}}$$

Table S1 Predicted micropore parameters of LMMAP for figs preservation under ambient temperature conditions

Micropores (number)	10	20	30	40	50
Diameters (μm)	383.36	304.21	265.25	241.45	224.13

Table S2 Criteria for sensory evaluation of figs

Indicators	Evaluation criteria	Score
Appearance	Rosy color, no rot, no browning	8-10
	Dark color, no gloss, no rot	5-7
	Dark red color, slight rot, moderate browning	3-4
	Severe browning and rot	1-2
Texture	Good firmness, no shriveling	8-10
	Good firmness, slight soft and wrinkled fruit	5-7
	Soft and shriveled fruit	3-4
	Severely soft fruit, juice flowing from the bottom	1-2
Flavor	Rich, sweet, fruity aroma	8-10
	Slight faint aroma	5-7
	Slight alcoholic or sour taste	3-4
	Alcoholic or moldy smell	1-2
Flesh quality	No browning, small cavity area	8-10
	Mild browning, small cavity area	5-7
	Browning, slight shriveled flesh, relatively large cavity area	3-4
	Severe browning and shriveled flesh, large cavity area	1-2
